# Enhancement of Partial Nitrification–Anaerobic Ammonia Oxidation in SBR Reactors via Surface-Modified Polyurethane Sponge Biofilm Carrier

**DOI:** 10.3390/polym17091145

**Published:** 2025-04-23

**Authors:** Zexiang Liu, Zhihong Xu, Kelin Li, Li Xie, Biao Han, Qiming Wang, Hainong Song, Jian Zhang

**Affiliations:** 1 Guangxi Key Laboratory of Clean Pulp & Papermaking and Pollution Control, School of Light Industry and Food Engineering, Guangxi University, Nanning 530004, China; 2 Scientific Research Academy of GuangXi Environmental Protection, Nanning 530022, China; 3 Guangxi Bossco Environmental Protection Technology Co., Ltd., Nanning 530007, China

**Keywords:** partial nitrification–anammox, sequencing batch reactor, zeolite

## Abstract

The partial nitrification–anammox process offers a cost-effective, energy-efficient, and environmentally sustainable approach for nitrogen removal in wastewater treatment. However, its application under low ammonia nitrogen conditions faces operational challenges including prolonged start-up periods and excessive nitrite oxidation. This study employed a strategy combining polyurethane surface positive charge enhancement and zeolite loading to develop a carrier capable of microbial enrichment and inhibition of nitrate generation, aiming to initiate the partial nitrification-anammox process in a sequencing batch reactor. Operational results demonstrate that the modified carrier enabled the reactor to achieve a total nitrogen removal efficiency of 78%, with the effluent nitrate nitrogen reduced to 6.03 mg-N/L, successfully initiating the partial nitrification-anammox process. The modified carrier also exhibited accelerated biofilm proliferation (both suspended and attached biomass increased). Additionally, 16S rRNA revealed a higher relative abundance of typical anammox bacteria *Candidatus* Brocadia in the biofilm of the modified carrier compared to the original carrier, alongside a decline in nitrifying genera, such as *Nitrolancea*. These microbial shifts effectively suppressed excessive nitrite oxidation, limited nitrate accumulation, and sustained efficient nitrogen removal throughout the reactor’s operation.

## 1. Introduction

Biological nitrogen removal technology has a development history of nearly a century [1]. Conventional approaches relying on nitrification–denitrification processes remain energy- and resource-intensive, demanding significant aeration and external carbon supplementation while generating substantial operational costs [2]. Anaerobic ammonium oxidation (anammox) utilizes anaerobic ammonium-oxidizing bacteria (AnAOB) to directly oxidize ammonium into gaseous nitrogen (N_2_), requiring only minimal aeration and no external carbon source [3]. AnAOB carry out the anaerobic ammonium oxidation reaction within the unique organelle (the anammoxosome), using ammonium nitrogen as the electron donor and nitrite nitrogen as the electron acceptor [4,5]. As an effective and environmentally friendly technology, this method has attracted extensive attention and is used to treat ammonia nitrogen-rich wastewater.

The anammox process requires co-existing ammonium (NH_4_^+^-N) and nitrite (NO_2_^−^-N) substrates for its biochemical reactions, yet most wastewaters contain only ammonium. This fundamental limitation requires controlled partial ammonia oxidation to nitrite as the critical precursor for anammox-mediated nitrogen removal [6,7]. To address this substrate requirement, the partial nitrification–anammox (PN/A) process has been developed as an integrated solution for nitrogen-laden wastewater treatment [8]. The PN/A process is a two-step process where ammonia-oxidizing bacteria (AOB) first oxidize ammonia nitrogen to nitrite under aerobic conditions, followed by anaerobic ammonium-oxidizing bacteria (AnAOB) converting the remaining ammonia nitrogen and nitrite to N_2_ under anaerobic conditions, thereby achieving nitrogen removal [9,10].

The PN/A process achieves efficient nitrogen removal through autotrophic ammonium conversion under limited aeration, offering significant economic and environmental advantages by eliminating external carbon requirements [11]. The prerequisite for successfully initiating the PN/A process is retaining a sufficient quantity of microorganisms in the reactor and constructing a microbial community with functionally compatible populations [12]. Key operational challenges include the following: (1) Members of the AnAOB grow slowly, with a long doubling time (12 days) [13]. Therefore, since AnAOB grow slowly, it is necessary to retain AnAOB in the reactor in the form of biofilms or granules to prevent biomass washout [14]. (2) The target product nitrite may be further oxidized by nitrite-oxidizing bacteria (NOB) to nitrate [10]. Nitrate cannot be removed in the absence of organic carbon sources in wastewater, leading to decreased total nitrogen removal efficiency. To address these challenges, the following two critical optimization strategies emerge: (1) enhancing microbial enrichment and improving the activity of anammox bacteria; (2) selectively inhibiting the activity of nitrite-oxidizing bacteria to prevent excessive nitrite oxidation [15].

The sequencing batch reactor (SBR) enables operational optimization through adjustable aeration periods for sedimentation control and hydraulic selection-based biomass retention [15]. However, hydraulic selection formed by controlling SBR settling duration cannot fundamentally regulate the AOB/NOB ratio. Improper control inevitably causes excessive ammonia oxidation, leading to effluent nitrate accumulation. Since AOB exhibit a stronger affinity for dissolved oxygen (DO) than NOB, intermittent aeration can be adopted to suppress NOB activity, preventing excessive nitrite oxidation [16]. Furthermore, since NOB are highly sensitive to free ammonia (FA) concentrations, establishing localized ammonia-rich zones can strategically inhibit NOB metabolic activity [17].

Sludge granulation and biofilm formation are the main means to meet the prolonged sludge age required for AnAOB enrichment. Under low ammonia nitrogen concentration conditions, granules are prone to disintegration, while introducing carriers to form biofilms provides higher stability, enabling tolerance to environmental fluctuations and hydraulic shear [18]. Polyurethane sponges are regarded as ideal microbial immobilization carriers due to their high porosity (facilitating substrate diffusion), large specific surface area, and fluidization capability. Furthermore, their interface design and modification can create interfacial microenvironments initiated by cell adhesion and microbial selection, accelerating PN/A process initiation. First, by introducing positively charged polyquaternium-10, the positive charge on the carrier surface is enhanced, promoting initial microbial attachment and biofilm formation [19]. Second, considering the sensitivity of NOB to free ammonia (FA), zeolite, with excellent ion adsorption and exchange capacity, can be incorporated onto the carrier surface [11]. By endowing the carrier with a dynamic buffering capacity for local ammonia nitrogen concentration, NOB-mediated side reactions are suppressed, improving the conversion efficiency of ammonia nitrogen to N_2_ via the PN/A process pathway. Compared with the common polyurethane-modified carriers, the carrier developed in this study has higher stability, stronger resistance to environmental shocks, and due to its surface modification, facilitates easier biofilm formation and a faster start-up of the PN/A process.

This study employed a composite carrier with a positively charged surface modification and zeolite loading for microbial retention. By modifying the carrier surface with a positive charge potential, microbial adsorption was enhanced, promoting biofilm formation. Through the carrier modification of the local microenvironment, NOB growth was inhibited, while the activity of AOB and AnAOB was strengthened. The investigation focused on nitrogen removal efficiency during reactor start-up, morphological changes in sludge flocs and biofilms across operational phases, and shifts in the microbial community structure.

## 2. Materials and Methods

### 2.1. Material

#### 2.1.1. Seed Sludge

The nitrifying activated sludge used in the experiment was sourced from the wastewater treatment reactor of Angel Yeast (Chongzuo) Co., Ltd., Chongzuo, China The retrieved granular sludge was mechanically disintegrated into flocculent sludge and mixed with anaerobic ammonium oxidation (anammox) flocculent sludge from the laboratory at a ratio of 2:1.

#### 2.1.2. Synthetic Wastewater

To stimulate the activity of aerobic nitrifying bacteria, the chemical oxygen demand (COD) concentration was maintained at 100 mg per liter in the form of glucose during the initial ten days of the experiment [20].

Other influent substrates for the simulated effluent included 21.9 mg/L potassium dihydrogen phosphate (KH_2_PO_4_), 36 mg/L calcium chloride monohydrate (CaCl_2_·H_2_O), and 25 mg/L magnesium sulfate hexahydrate (MgSO_4_·6H_2_O). The pH value of the simulated wastewater was adjusted to 8.0–8.5 by adding appropriate amounts of potassium hydroxide (KOH) and hydrochloric acid (HCl), and 1 mL of trace element I and trace element II were added to each liter of wastewater [21]. The compositions of these elements are shown in Table 1.

### 2.2. Methods

#### 2.2.1. Carrier Preparation and Reactor Description

**Figure 1:**
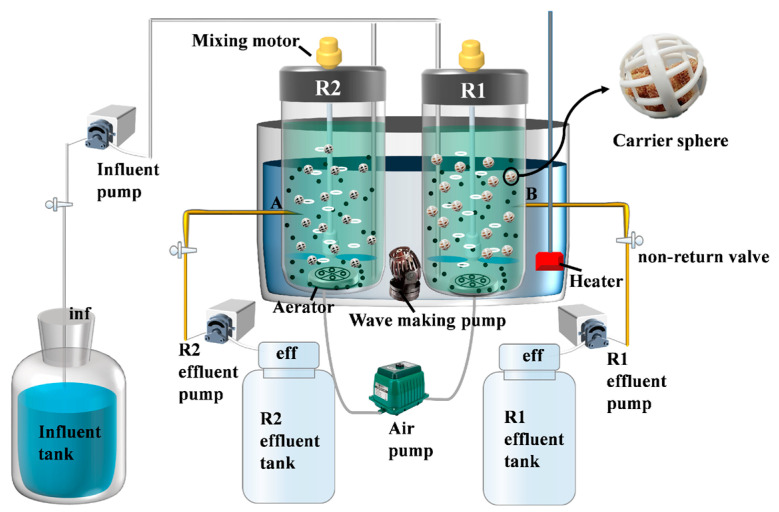
Schematic diagram of SBR reactors integrated with sponge carriers.

The composite carrier was synthesized using the impregnation method. A predetermined amount of polyvinyl alcohol (PVA) was dissolved in deionized water at 95 °C to prepare a 10% (*w*/*v*) PVA solution. This solution was mixed with a 5% (*w*/*v*) polyquaternium-10 solution at a volume ratio of 10:1, stirred for 20 min to form a homogeneous solution. Zeolite powder and water-based polyurethane were blended at a weight ratio of 27:40, followed by the addition of the PVA-polyquaternium-10 solution. The mixture was stirred for 1 h to ensure thorough homogenization. A suitable number of 1 cm^3^ polyurethane foam carriers were fully immersed in the homogeneous solution. After immersion, the carriers were rinsed three times with deionized water, dried, and stored in vacuum-sealed bags [22]. Each carrier contained approximately 0.06 g of zeolite. The experimental system employed a sequencing batch reactor (SBR), with its operational configuration schematically detailed in Figure 1. In the figure, R1 is the reactor with an unmodified carrier (polyurethane sponge not prepared by the impregnation method) and R2 is the reactor with a modified carrier (composite carrier prepared by the impregnation method).

#### 2.2.2. Reactor Operation and Control

The reactor was operated for 116 days. The whole operation period can be divided into three phases: the start-up period (Phase I: 1–20 d), the transition period (Phase II: 21–80 d), and the stabilization period (Phase III: 81–116 d). The specific parameters of the reactor are shown in Table 2.

#### 2.2.3. Sludge Activity Detection

Measurement of biofilm loading on the carrier [23]:

An appropriate number of carriers was removed from the reactor and placed in a 250 mL beaker. Then, 50 mL of deionized water was added. The mixture was stirred on a magnetic stirrer at 300 r/min for 1 h. The carriers were removed, squeezed to drain excess water, and returned to the reactor. The obtained suspension was tested for mixed liquor suspended solids (MLSS) and mixed liquor volatile suspended solids (MLVSS) using standard methods. The biofilm loading on the carriers, expressed as attached growth biomass solids (AGBS) and volatile attached growth biomass solids (VAGBS), was calculated by dividing the MLSS and MLVSS by the number of carriers removed.

Measurement of activated sludge particle size [24]:

Three biofilm carriers were randomly selected from the reactor. After ultrasonic treatment to detach the sludge from the carriers, the carriers were returned to the reactor. The obtained suspension was dispersed using deionized water, and the particle size of the activated sludge was measured using a laser particle size analyzer. This procedure was repeated three times, and the average value was recorded.

#### 2.2.4. Water Quality Detection

The reactor influent and effluent samples were monitored daily, including NH_4_^+^-N (ammonia nitrogen), NO_2_^−^-N (nitrite nitrogen), NO_3_^−^-N (nitrate nitrogen), and pH [25]. The ammonia nitrogen was detected by Nano reagent spectrophotometry, the nitrite nitrogen was detected by UV spectrophotometry, and the nitrate nitrogen by the thymol method [13]. The effluent parameters were tested daily during the first 10 days of operation and subsequently every 2 days (no statistical significance test was conducted).

The nitrogen loading rate (NLR, in kg-N/m^3^·d^−^^1^), the total nitrogen removal rate (TNRE, in %), the ammonia removal rate (ARE, in %), the ammonia removal rate (ARR, in kg-N/m^3^·d^−^^1^), the ammonia oxidation rate (AOR, in kg-N/m^3^·d^−^^1^), the nitrite oxidation rate (NOR, in kg-N/m^3^·d^−^^1^), and the total nitrogen removal rate (TNRR, unit: kg-N/m^3^·d^−^^1^) were calculated as shown in Equations (1)–(6), and the ∆ NO_3_^−^-N/∆ NH_4_^+^-N in the PN/A was calculated as shown in Equation (7). The inf subscripts denote the influent water, and the eff subscripts denote the effluent water.

(1) $$NLR=\left(\frac{{C\left(TN\right)}_{inf}}{HRT}\right)\times 24\times 10^{-3}$$

(2) $$TNRE=\frac{{C\left(TN\right)}_{inf-{C\left(TN\right)}_{eff}}}{{C\left(TN\right)}_{inf}}\times 100$$

(3) $$ARE=\frac{{C\left({NH}_{4}^{+}-N\right)}_{inf-}{C\left({NH}_{4}^{+}-N\right)}_{eff}}{{C\left({NH}_{4}^{+}-N\right)}_{eff}}\times 100$$

(4) $$AOR=\left(\frac{{C\left({NH}_{4}^{+}-N\right)}_{inf-}{C\left({NH}_{4}^{+}-N\right)}_{eff}-\frac{∆C\left(TN\right)}{2.04}}{HRT}\right)\times 24\times 10^{-3}$$

(5) $$NOR=\left(\frac{{C\left({NO}_{3}^{-}-N\right)}_{eff}-\frac{0.26∆C\left(TN\right)}{2.04}}{HRT}\right)\times 24\times 10^{-3}$$

(6) $$TNRR=\frac{∆C\left(TN\right)}{HRT}\times 24\times 10^{-3}$$

(7) $$\frac{{∆NO}_{3}^{-}}{{∆NH}_{4}^{+}}=\frac{{C\left({NO}_{3}^{-}-N\right)}_{eff-}{C\left({NO}_{3}^{-}-N\right)}_{inf}}{{C\left({NH}_{4}^{+}-N\right)}_{inf-}{C\left({NH}_{4}^{+}-N\right)}_{eff}}$$

#### 2.2.5. Extracellular Polymeric Substances (EPS) Extraction and Speciation

The improved total EPS extraction method was as follows: First, 5 mL of sludge was taken and washed three times with ultrapure water. The washed sludge was resuspended in 15 mL of 0.01 M PBS buffer (pH = 7.2) and centrifuged at 7000× *g* rpm for 20 min. NaOH was added to adjust the mixture to pH = 13. The supernatant was filtered through a 0.45 μm PES membrane to obtain the total EPS. The three subcomponents of the EPS were extracted using a modified heat method [26].

S-EPS: The washed 5 mL sludge was mixed with 15 mL PBS and centrifuged at 2000× *g* rpm for 1 min. The supernatant was discarded, and the remaining sludge was mixed with 15 mL of 0.05% NaCl solution, followed by centrifugation at 4000× *g* rpm for 5 min. The supernatant was filtered through a 0.45 μm PES membrane.

LB-EPS: Preheated 0.05% NaCl solution (60 °C) was added to the remaining sludge to adjust the total volume to 20 mL. After vortex mixing for 2 min, the mixture was centrifuged at 4000× *g* rpm for 10 min. The supernatant was collected by filtration through a 0.45 μm membrane.

TB-EPS: The remaining sludge was resuspended in 15 mL of 0.05% NaCl solution and heated in a water bath at 60 °C for 30 min. Following vortex mixing for 2 min and centrifugation at 4000 rpm for 15 min, the supernatant was collected through a 0.45 μm membrane [27].

The extracted EPS was characterized by three-dimensional excitation emission matrix (3D-EEM) fluorescence spectroscopy [28].

The protein content was analyzed using the modified Lowey method, and the polysaccharide content was determined using the phenol–sulfuric acid method [29].

#### 2.2.6. Microbial Detection

Microbial community structure detection: Sludge samples were collected on days 20 and 100, respectively. Raw sludge and sludge samples from different reactors were labeled as Blank, R1, and R2. An appropriate amount of sludge was shipped under refrigeration to Majorbio BioPharm Technology Co., Ltd., Shanghai, China for high-throughput sequencing. The key steps of the high-throughput sequencing were as follows: DNA was extracted from the sludge using the E.Z.N.A.^®^ Mag-Bind Soil DNA Kit (OMEGA), and bacterial and archaeal sequences were amplified using primers 52F10extF-Arch958RmodR and 338F-806R, respectively. PCR products were detected via 2% agarose gel electrophoresis, purified with the AxyPrep DNA Gel Extraction Kit, and sequenced on the Illumina MiSeq platform to generate a PE amplicon library. Operational taxonomic units (OTUs) were classified and analyzed [12].

## 3. Results and Discussion

### 3.1. Characterization of Modified Polyurethane Sponge Carrier

#### 3.1.1. Surface Properties

**Figure 2:**
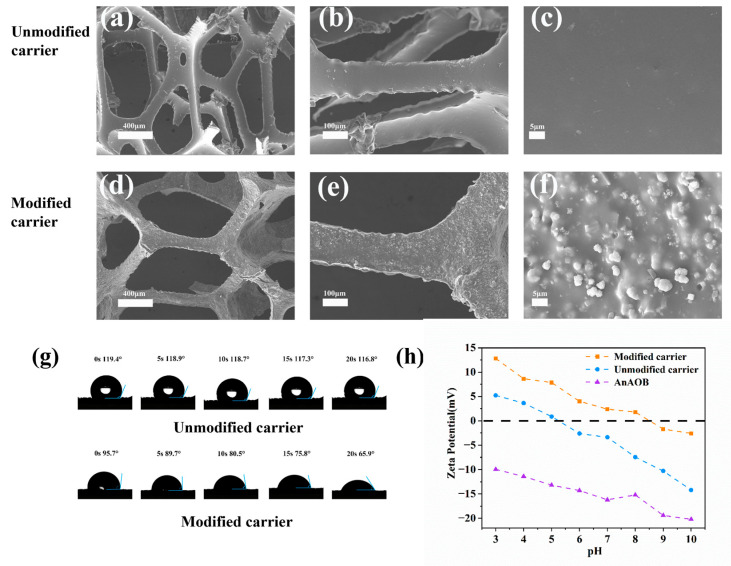
Modification of surface morphology (**a**–**f**), contact angle (**g**), and zeta potential (**h**).

Figure 2a–f present a comparative SEM characterization of the carrier surface morphology. Due to the zeolite loading, the composite carrier exhibited increased surface roughness, which aided in resisting hydraulic shear during the initial microbial adhesion stage, enriching microorganisms, and accelerating biofilm formation. Figure 2g displays the water contact angles of the carrier before and after modification. The modified carrier transitioned from hydrophobic to hydrophilic due to the incorporation of hydrophilic materials, polyvinyl alcohol (PVA) and zeolite, during fabrication [30]. Figure 2h illustrates the zeta potentials of the carrier before and after modification and the sludge biofilm. The composite carrier’s positive potential increased from −7.45 mV to 1.79 mV owing to the doping of the positively charged material polyquaternium-10, while the anammox sludge showed a negative charge [31]. This charge complementarity enhanced electrostatic microbe–carrier interactions, achieving faster biofilm formation than unmodified carriers [23].

#### 3.1.2. Ammonia Nitrogen Adsorption Capacity

**Figure 3:**
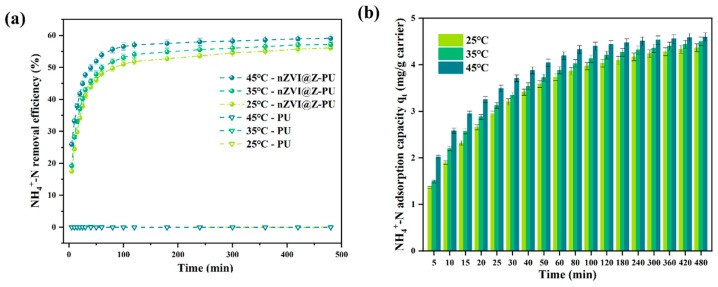
Kinetics of NH_4_^+^-N adsorption by the composite carrier at 35 °C (**a**); NH_4_^+^-N removal quantity before and after carrier modification (**b**).

Figure 3a illustrates the NH_4_^+^-N adsorption performance of the modified carriers. The composite carrier exhibited an initial increase followed by stabilization in ammonia nitrogen removal, consistent across different temperatures, whereas the unmodified carrier showed no adsorption capacity. This is attributed to the zeolite-loaded modified carrier adsorption capability [32].

As shown in Figure 3b, the NH_4_^+^-N adsorption capacity of the composite carrier displayed dynamic characteristics, with rapid initial adsorption followed by gradual stabilization. The maximum adsorption capacity reached 4.5028 mg/g carrier after 8 h.

### 3.2. Reactor Performance Enhanced by Carrier Modification

#### 3.2.1. Nitrogen Removal

**Figure 4:**
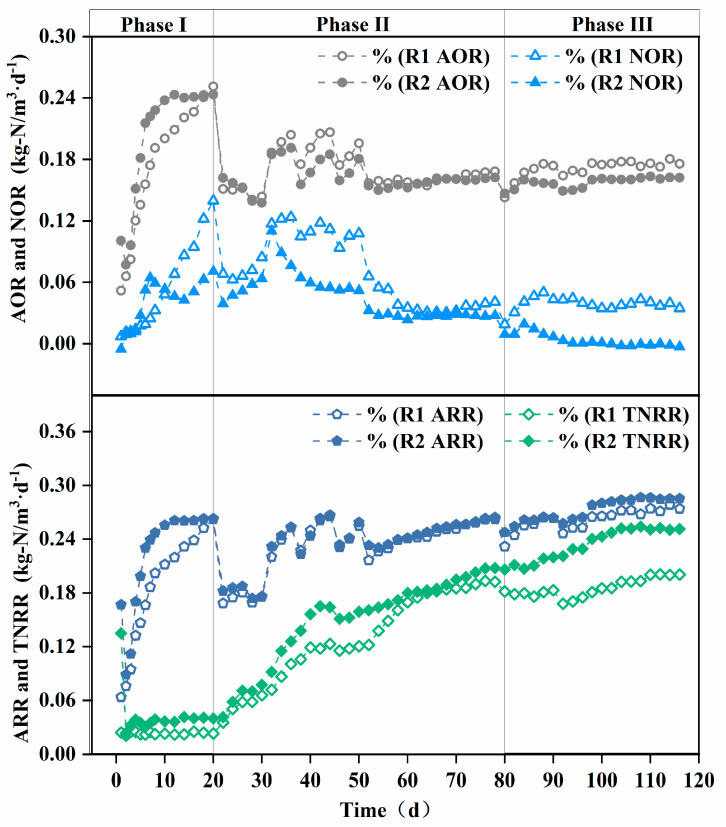
Enhancement of nitrogen removal in terms of AOR, NOR, ARR, and TNRR achieved by carrier modification. AOR: ammonia oxidation rate, NOR: nitrite oxidation rate, ARR: ammonia removal rate, TNRR: total nitrogen removal rate.

AOR, NOR, and TNRR indirectly reflect the theoretical in situ activities of the corresponding nitrifying microorganisms [33]. As shown in Figure 4, the AOR of both reactors gradually increased in Phase I, with the modified AOR being higher than the unmodified one. This is attributed to the modified carrier’s superior initial microbial adsorption and ammonia-rich microenvironment construction capabilities, facilitating the enrichment of AOB.

Sustained aeration and nitrite accumulation provided favorable conditions for NOB growth [34], leading to an increase in NOR and the recovery of NOB activity. However, the modified carrier achieved localized ammonia nitrogen inhibition, preventing a continuous NOR rise in the modified reactor.

The TNRE remained relatively low in both the modified and unmodified systems. In PN/A processes, total nitrogen removal primarily relies on AnAOB, which are inhibited under full aeration conditions [11]. Nevertheless, the TNRR of the reactor with the modified carrier was higher than that of the reactor with the unmodified carrier, due to the modified carrier’s enhanced initial microbial adsorption.

Phase II initiation triggered abrupt ARR/AOR declines in both reactors due to the oxygen-limited suppression of AOB activity. Meanwhile, the gradual increase in the TNRR indicates that the low-oxygen environment led to AnAOB growth, though excessive ammonia accumulation limited total nitrogen removal. To sustain AOB and AnAOB activity, a “graded aeration reduction strategy” was adopted from day 30. ARR and AOR fluctuated upward during Phase II, demonstrating AOB’s low-oxygen adaptability under fluctuating oxygen stress, while the steady rise in the TNRR reflects the continuous enhancement of AnAOB activity. Divergent NOR trends between the modified and unmodified carriers highlighted NOB as the primary factor influencing effluent nitrate differences [35]. The composite carrier’s ammonia-rich microenvironment progressively eliminated NOB, exhibiting strong NOB suppression [36].

**Figure 5:**
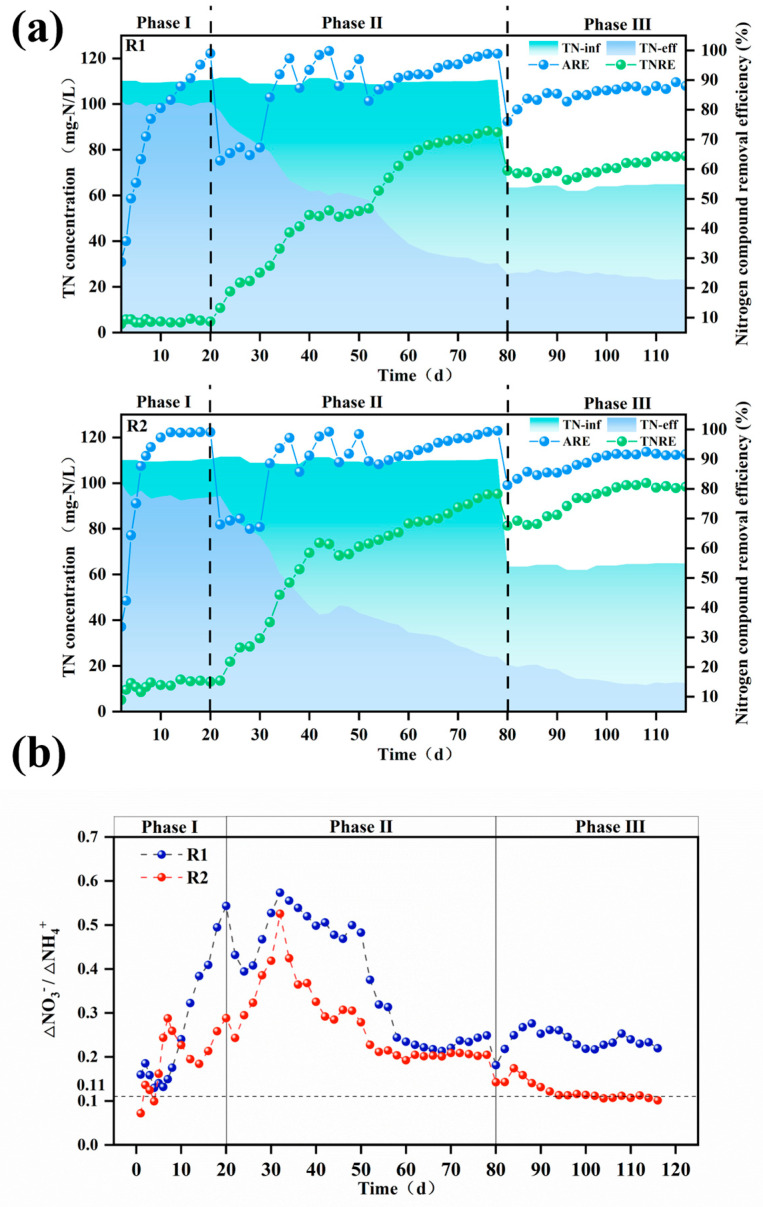
The TN, ARE, and TNRE of the reactor with unmodified (R1) and modified (R2) carriers (**a**); ∆NO_3_^−^-N/∆NH_4_^+^-N ratio throughout the operational period for both reactors (**b**). TN: total nitrogen, ARE:. ammonia removal efficiency, TNRE: total nitrogen removal efficiency.

As shown in Figure 4, reactor R2 with modified carriers exhibited a significantly higher total nitrogen removal rate (TNRR) (0.25 kg-N/m^3^·d^−^^1^ vs. 0.20 kg-N/m^3^·d^−^^1^) compared to the control reactor R1 in Phase III, while the ammonia removal rate (ARR) remained similar between the two reactors. This observation suggests that ammonia nitrogen in R1 may not have been sufficiently converted into nitrate for utilization in anaerobic ammonium oxidation (anammox). Since nitrate cannot be removed via denitrification in the absence of a carbon source, this limitation likely contributed to the lower TNRR in R1 compared to R2. These findings are corroborated by the reactor effluent data. As illustrated in Figure 5b, the ΔNO_3_^−^-N/ΔNH_4_^+^-N ratio in the modified carrier reactor was significantly lower than that in the unmodified carrier reactor (0.11 vs. 0.22). This confirms that the modified carrier enhanced the selectivity of ammonia oxidation, effectively suppressing nitrate formation and accumulation. Consequently, the modified carrier facilitated efficient ammonia nitrogen removal via anammox, ensuring stable reactor performance.

#### 3.2.2. Start-Up of PN/A Process

As shown in Figure 5a,b, it was observed that by the end of Phase I, both modified and unmodified systems achieved ammonia nitrogen removal efficiencies exceeding 95%, but the total nitrogen removal performance remained minimal. Upon reducing aeration in Phase II, ammonia removal rates experienced a trough, while the ∆NO_3_^−^-N/∆NH_4_^+^-N ratio gradually increased, surpassing the PN/A reaction’s standard value of 0.11. After implementing the “graded aeration reduction” strategy, ammonia removal rates underwent repeated fluctuations of increase–decrease–increase. By day 52, the modified and unmodified systems reached ammonia removal rates of 81% and 89%, respectively, indicating restored AOB activity. Concurrently, the effluent total nitrogen in both reactors progressively declined, with total nitrogen removal efficiencies reaching 46.7% and 61.5% for R1 and R2. Moreover, the ∆NO_3_^−^-N/∆NH_4_^+^-N ratio of modified and unmodified systems decreased from peak values of 0.57 and 0.52 to 0.25 and 0.20 by the end of Phase II, demonstrating significant overall performance improvement.

In Phase III, fluctuations in the ammonia removal rates occurred due to a reduced influent ammonia concentration and shorter hydraulic retention time (HRT). As the reaction progressed, microbial synergy strengthened continuously. The modified carrier successfully enriched functional microorganisms while suppressing NOB, leading to the gradual washout of non-functional bacteria from the reactor. By day 98, the ∆NO_3_^−^-N/∆NH_4_^+^-N ratio of the modified system reached 0.12, approaching the PN/A standard value of 0.11. At this stage, the total nitrogen removal efficiency was 78%, the ammonia removal efficiency was 90%, and the TNRR was 0.24 kg-N·m^−^^3^·d^−^^1^, successfully achieving PN/A initiation under low ammonia nitrogen conditions. In contrast, the unmodified carrier failed to initiate PN/A.

### 3.3. Biofilm and Microbial Community

#### 3.3.1. Biofilm and Biomass Growth on Carriers

**Figure 6:**
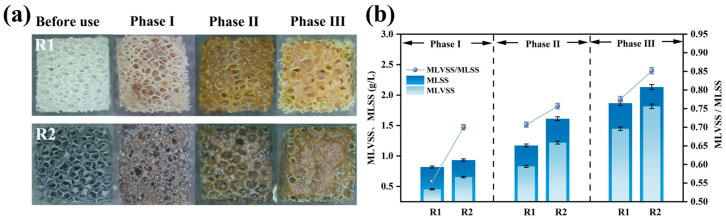
Biofilm growth on polyurethane sponge carrier: morphology of biofilm covering carrier surface (**a**); biofilm quantified by MLSS and MLVSS (**b**).

Figure 6a illustrates the biofilm growth on the carriers. Sludge attachment was observed to follow the sponge skeleton framework, progressing gradually from the interior to the exterior. Upon reaction completion, the modified carrier formed a thick sludge biofilm, with significantly greater surface biomass accumulation compared to the unmodified carrier [37]. This was due to the electropositive surface of the modified carrier enhancing microbial adsorption. Sludge coverage reduced the oxygen penetration capacity in the modified carrier compared to the unmodified one. Owing to the porous structure of the polyurethane sponge, microorganisms continued to colonize the internal spaces of the carrier. Increased microbial attachment led to thicker biofilms, facilitating the development of anoxic zones within the carrier matrix, thereby promoting anammox and enhancing nitrogen removal [8]. This also explains the higher ammonia removal efficiency of the modified carrier.

The MLVSS/MLSS ratio reflects the proportion of active biomass in the sludge biofilm [38]. As shown in Figure 6b, the MLSS and MLVSS in R2 (modified) remained higher than in R1 (unmodified). This phenomenon stems from the modified carrier’s positive charge improving its initial microbial adsorption. During operation, suspended floccular sludge was continuously washed out, while microbial aggregates adhered to the carrier, resisting hydraulic shear and avoiding discharge, leading to gradual biomass accumulation.

The MLVSS/MLSS ratios for the modified and unmodified systems increased from 0.55 and 0.70 in Phase I to 0.77 and 0.85 in Phase III, respectively. The higher MLVSS/MLSS ratio in the modified system indicates greater biological activity in its biofilm. At the end of the reaction, the MLVSS reached 1.82 g/L for the modified system versus 1.45 g/L for the unmodified system. Due to oxygen concentration gradients, AnAOB preferentially thrive in biofilms; therefore, higher MLVSS confirm the enhanced nitrogen removal capacity of the modified carrier [14].

Post-reaction, the MLSS, MLVSS, attached granular biomass (AGBS), and volatile attached granular biomass (VAGBS) on individual carriers in the modified and unmodified reactors were quantified (Table 3).

Both the MLSS and AGBS demonstrated superior performance in the modified reactor compared to the unmodified. The modified carrier exhibited a VAGBS of 7.92 ± 0.01 mg/carrier, 1.45-fold higher than the unmodified carrier’s 5.45 ± 0.01 mg/carrier. The greater biomass in the modified reactor further verifies that the composite carrier promoted microbial adsorption more than the original carrier. The modified carrier effectively retained microorganisms under hydraulic scouring, enabling faster biofilm growth compared to the unmodified carrier [37]. Higher microbial abundance on the carrier accelerated metabolic rates, resulting in increased oxygen consumption.

#### 3.3.2. EPS Composition and Speciation

**Figure 7:**
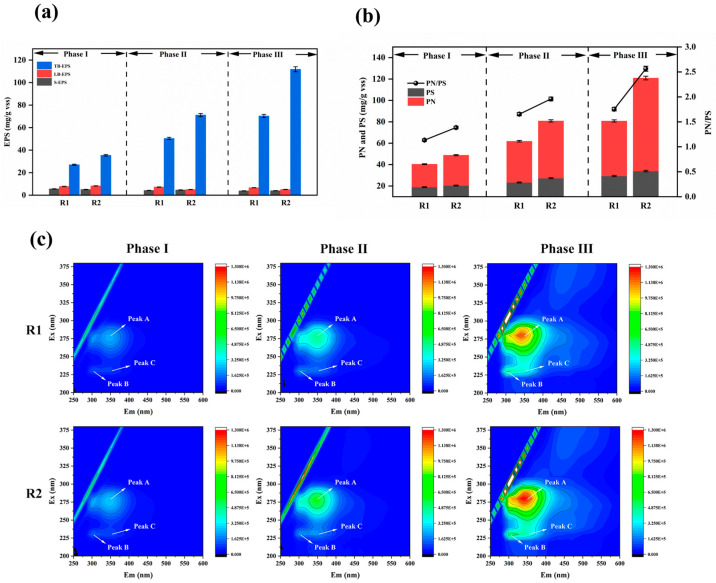
Growth of EPS on carriers during initiation of partial nitrification–anaerobic ammonia oxidation (**a**); PN and PS and their ratios in EPS on carriers during partial nitrification–anaerobic ammonia oxidation initiation at each phase (**b**); EEM spectra of TB-EPS on carriers during PN/A initiation (**c**).

Extracellular polymeric substances (EPS) are crucial components of biofilms, contributing to matrix structure integrity and stability and the protection of embedded microorganisms against toxic effects [39]. EPS exhibit distinct stratified structures in biofilms, including soluble EPS (S-EPS), which weakly associate with microbial aggregates or dissolve in the surrounding solution, forming a discrete overlayer with distinct boundaries outside the cell walls; loosely bound EPS (LB-EPS); and tightly bound EPS (TB-EPS), which collectively form a bilayer structure. All EPS fractions are enriched with negatively charged groups, with TB-EPS constituting the predominant proportion [40]. Figure 7a compares the concentrations and proportions of EPS fractions in the modified and unmodified reactors.

Experimental observations revealed minimal variation in the S-EPS and LB-EPS levels, with their contents in the sludge biofilms being significantly lower than TB-EPS. Therefore, EPS dynamics can primarily be attributed to TB-EPS changes. The TB-EPS correlated closely with the water quality and sludge morphology, serving as an indicator of microbial viability and aggregate structure. In the unmodified reactor, the TB-EPS increased from 27.03 mg/g VSS in Phase I to 70.34 mg/g VSS in Phase III, representing a 160% rise (43.31 mg/g VSS increment). In contrast, the modified reactor exhibited a more substantial TB-EPS increase from 35.44 mg/g VSS in Phase I to 111.83 mg/g VSS in Phase III, a 216% surge (76.39 mg/g VSS increment). EPS enhance biofilm adhesiveness, promote microbial aggregation and growth, and improve biofilm attachment [41]. The abundant EPS production accelerated biofilm formation on the modified carriers, increasing oxygen consumption and AnAOB activity, thereby enhancing ammonia removal efficiency [42].

EPS primarily comprise extracellular polysaccharides (PS) and proteins (PNs) [43]. The data of Figure 7b show that the PS content remained consistently lower than the PNs across all experimental phases in both the modified and unmodified systems. While the PS levels showed negligible differences between the modified and unmodified carriers, substantial PN disparities were observed, indicating that higher TB-EPS in the modified system predominantly resulted from increased PN accumulation. A higher PN/PS ratio reflects enhanced biofilm hydrophobicity. Increased sludge hydrophobicity is critical for microbial aggregation and biofilm development.

In Phase I, the PN/PS ratios for the modified and unmodified systems were 1.13 and 1.39, respectively, with PS values of 18.94 mg/g VSS and 23.32 mg/g VSS, showing minor differences. This is because during the initial adhesion stage, biofilms require increased PS secretion to form a buffer layer between the aqueous environment and microorganisms, protecting them from trace toxic substances and adapting to relatively harsh conditions. Meanwhile, PS enhance the compactness and stability of biofilm structures. EPS form gels via hydrogen bonds, and the PS content in EPS plays a critical role in the initial microbial attachment [44].

During Phases II and III, the PN/PS ratios in the modified reactor remained higher than those in the unmodified reactor. The increasing disparity reflects dynamic changes in the PN and PS compositions across reaction phases. The strong aggregation capacity of activated sludge is primarily attributed to PNs, which is dominated by hydrophobic amino acids. The hydrophobicity of the PNs correlates positively with EPS hydrophobicity, thereby promoting flocculation [45].

Figure 7c presents the EEM spectra of sludge TB-EPS from both reactors at different operational phase, revealing three distinct EPS-associated fluorescence peaks. Peak A (275 nm/340–360 nm) corresponds to tryptophan in proteins, Peak B (230 nm/300–330 nm) represents tyrosine, and Peak C (220 nm/335–355 nm) reflects aromatic protein-like substances. The peak positions show negligible shifts, indicating no significant changes in the EPS chemical composition. The fluorescence intensities gradually increased with the reaction’s progression, aligning with the PN trends in the EPS. As the particle size and biofilm thickness increased during the operation, EPS secretion intensified. The fluorescence intensities of Peaks A and B rose significantly, with tryptophan becoming more prevalent during biofilm development, serving structural and stabilizing roles in EPS. Tryptophan fluorescence properties, monitored via EEM, aid in studying EPS composition and dynamics. The modified system exhibited higher intensity ratios of tryptophan-to-tyrosine peaks compared to the unmodified reactor (R1), reflecting enhanced protein synthesis and accumulation in the modified biofilms. The elevated PN/PS ratios increased the sludge surface’s hydrophobicity, accelerating biofilm formation and improving structural robustness [46].

#### 3.3.3. Microbial Community in Biofilm

**Figure 8:**
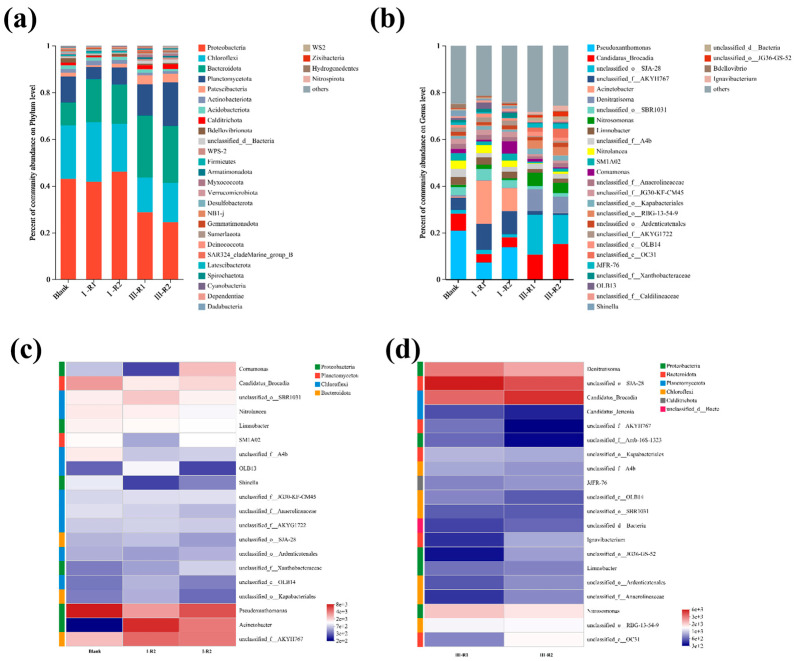
Evolutionary patterns at the phylum level of carrier microbial communities during PN/A initiation (**a**); evolutionary patterns at the genus level of vector microbial communities during PN/A initiation (**b**); heatmap depicting microbial abundance in seed sludge and various reactors during phase I (**c**); heatmap depicting microbial abundance in different reactors during phase III (**d**).

To explore microbial community evolution during the start-up of a single-stage PN/A process, high-throughput sequencing was performed on sludge samples from a blank control (inoculated sludge) and reactors with modified or unmodified carriers. Figure 8a shows the microbial community composition at the phylum level. The dominant phyla included *Proteobacteria*, *Chloroflexi*, *Bacteroidota*, and *Planctomycetota*. *Proteobacteria* encompasses AOB and NOB [47]. *Chloroflexi* contains minor NOB populations, while *Planctomycetota* primarily harbors anaerobic AnAOB [48]. Figure 8b displays genus-level taxonomic profiles. *Denitratisoma* spp. represents denitrifying bacteria. *Candidatus* Brocadia spp. and *Candidatus* Kuenenia spp. are typical AnAOB genera in wastewater systems [49]. *Nitrosomonas* spp. (AOB) and *Nitrolancea* spp. (NOB) were also identified.

As shown in Figure 8a, *Planctomycetota* abundances in the modified and unmodified reactors were lower than in the blank control. This may be attributed to the glucose supplementation only during the first 10 days of Phase I, which likely eliminated heterotrophic microorganisms within *Planctomycetota* under unfavorable conditions. Since *Planctomycetota* hosts AnAOB, increased dissolved oxygen from aeration inhibited their growth [2]. Notably, the modified reactor exhibited higher *Planctomycetota* abundance than the unmodified reactor, possibly due to localized ammonia-rich microenvironments around the modified carrier, enhancing AnAOB activity and ammonia removal.

By the end of Phase III, decreased *Proteobacteria* and *Chloroflexi* abundances in both reactors likely resulted from NOB suppression and partial AOB washout from flocs. Bacteroidota enrichment correlated with biofilm development, as its associated microorganisms promoted denser biofilm structures. The increased *Planctomycetota* proportions confirm AnAOB proliferation. Notably, the *Planctomycetota* abundance in the modified reactor was 1.4-fold higher than in the unmodified system, demonstrating the modified carrier’s superior ability to support AnAOB growth.

Figure 8b displays genus-level microbial community composition, revealing significant microbial shifts. The relative abundances of *Denitratisoma* spp. increased in both the modified and unmodified reactors during Phase III. This is likely because high aeration initially suppressed its activity, while reduced dissolved oxygen levels later provided favorable conditions for its recovery.

As shown in Figure 8c,d, the abundance of NOB was far lower than that of AOB, indicating that gradual aeration reduction successfully suppressed NOB activity, facilitated their washout, and effectively inhibited NOB proliferation. The enrichment of *Candidatus* Kuenenia spp. and *Nitrosomonas* spp. in the PN/A biofilm aligns with previous effluent results. This demonstrates that the gradual aeration reduction strategy maintained AOB activity while suppressing NOB activity and enhancing AnAOB activity. Higher proportions of *Candidatus* Kuenenia spp. and *Nitrosomonas* spp. in the modified reactor compared to the unmodified reactor suggest that the ammonia-rich microenvironment formed by the modified carrier promoted AnAOB activity improvement.

## 4. Conclusions

This study engineered a dual-functional polyurethane carrier incorporating surface charge modification (polyquaternium-10) and zeolite integration to optimize PN/A process initiation in SBR systems. By introducing the positively charged material polyquaternium-10, the carrier’s surface charge increased from −7.45 mV to +1.79 mV, facilitating biofilm formation on the carrier surface and increasing both suspended biomass and attached biomass. Zeolite loading endowed the carrier with an ammonia nitrogen adsorption capacity of 4.50 mg/g carrier. The detection results of the microbial community structure reveal decreased NOB abundance and increased AnAOB abundance, suggesting that the zeolite-modified surfaces created an ammonia-rich microenvironment that suppressed NOB activity while enhancing AnAOB activity. The reactor operation results demonstrate that the composite-modified carrier successfully initiated the PN/A process, whereas reactors with unmodified carriers failed to establish the PN/A process. During the operation of the experimental conditions, the modified carrier did not break. However, due to the relatively loose pores of the polyurethane sponge, the material had a certain service life and needed to be replaced regularly. The modified carrier has the ability to rapidly start-up PN/A and has a certain resistance to environmental shocks. It has a promising future in larger-scale applications.

## Figures and Tables

**Table 1:** Trace element composition in the stock solution.

Trace Element I	Concentration (g/L)	Trace Element II	Concentration (g/L)
2Na·EDTA·2H_2_O	15	2Na·EDTA·2H_2_O	5
Na_2_SeO_3_	0.156	FeSO_4_·7H_2_O	5
Na_2_MoO_4_·2H_2_O	0.22		
NiCl_2_·6H_2_O	0.19		
MnCl_2_·4H_2_O	0.99		
CuSO_4_·5H_2_O	0.25		
ZnSO_4_·7H_2_O	0.43		
CoCl_2_·6H_2_O	0.24		
H_3_BO_3_	0.014		

**Table 2:** Phase-specific parameters for the operation of both SBR reactors.

Stage	Time (d)	NH4^+^-N (mg N/L)	Air (L/min)	HRT (h)	NLR (kg-N/m^3^·d^−1^)
I Start-up period	1~20	110	0.15	10	0.264
II Transition period	21~78	110	0.04~0.1	10	0.264
III Stable period	79~116	60	0.04	5	0.288

**Table 3:** Suspended biomass and attached biomass on carriers.

	MLSS (g/L)	MLVSS (g/L)	AGBS (mg/Carrier)	VAGBS (mg/Carrier)
R1	0.82 ± 0.02	0.66 ± 0.05	6.83 ± 0.01	5.45 ± 0.01
R2	1.12 ± 0.06	0.95 ± 0.03	9.37 ± 0.01	7.92 ± 0.01
Growth	0.30 ± 0.04	0.29 ± 0.03	2.54 ± 0.01	2.47 ± 0.01

## Data Availability

The original contributions presented in this study are included in the article. Further inquiries can be directed to the corresponding authors.
